# Kaleidoscopic Incommensurate Structures of Aluminum-Substituted Rhenium Silicides

**DOI:** 10.1021/acs.inorgchem.5c01400

**Published:** 2025-06-23

**Authors:** Victoria Decocq, Fei Wang

**Affiliations:** Department of Chemistry and Biochemistry, 7471 Missouri State University, Springfield, Missouri 65897, United States

## Abstract

Al substitution leads to kaleidoscopic vacancy-bearing crystal structures in the rhenium silicides, ReAl_*x*_Si_1.75–0.75*x*_□_0.25–0.25*x*_ (□ indicates Si/Al vacancies), including two incommensurate structures newly found and successfully solved with (3 + 1)-dimensional superspace groups in this work. The similarity and difference among these structures, especially the subtle difference in the ordered distribution of vacancies, are compared and discussed in detail. With synthetic, crystallographic, and computational investigations, we gained a comprehensive understanding of the composition–structure relationship in these compounds, especially the ordered distribution of Si/Al vacancies.

## Graphical abstract

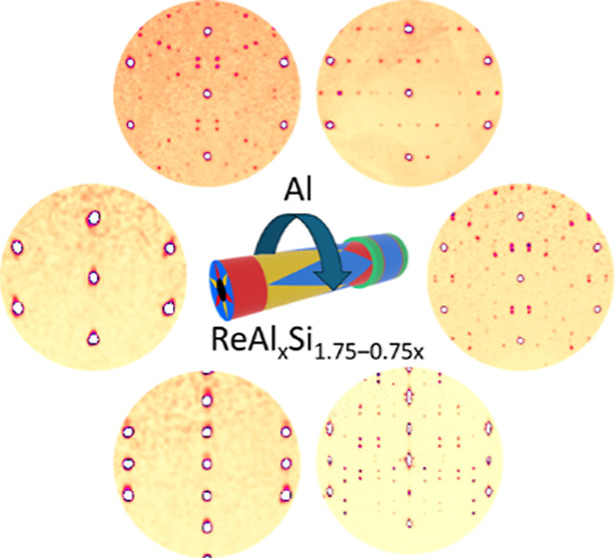

## Introduction

Rhenium and silicon form 3 binary intermetallic compounds: Re_2_Si, ReSi, and ReSi_1.75_ (or Re_4_Si_7_, which used to be incorrectly reported as ReSi_2_).^1,2^ Among them, ReSi_1.75_, along with its ternary variants, has been the most intensively studied for its complex crystal structure and intriguing physical properties.

The structure of ReSi_1.75_ itself had been reported in several space groups: *I*4/*mmm*,^3^ *Immm*,^4^ and *P*1.^5^ Later reports^6–8^ all reveal that it is actually a monoclinic (*Cm*) supercell/commensurate variant of the tetragonal (*I*4/*mmm*) MoSi_2_-type structure. Equivalently (and more efficiently using fewer parameters), the ReSi_1.75_ structure can also be depicted in the (3 + 1)-dimensional superspace group, *X*2/*m*(α0γ).^9^ Its complex crystal structure lends ReSi_1.75_ promising thermoelectric properties with a *ZT* value of 0.7 at 1073 K.^10^

What makes ReSi_1.75_ even more fascinating is that upon addition of a third element (such as Mo or Al) to substitute either Re or Si, its structure can be subtly tuned. For instance, at low-level substitution, ReAl_0.100_Si_1.675_ and Re_0.98_Mo_0.02_Si_1.73_ adopt aperiodic incommensurate structures, which also boast improved thermoelectric properties.^9,11–13^ Upon high-level Al substitution, ReAl_1.2_Si_0.8_ is isotypical to MoSi_2_ and exhibits superconductivity at ∼3.5 K.^14^

Compared to its parent structure, the MoSi_2_-type, ReSi_1.75_ can be viewed as ReSi_1.75_□_0.25_, where □ indicates Si vacancies. As shown in Figure 1, the vacancies occur in a specific way: two neighboring rows of Si along the ***b***-axis in the MoSi_2_-type structure are removed, and the Si atoms in the vicinity are displaced to compensate for the voids. These displaced Si atoms appear “flat” (highlighted in pink boxes in Figure 1) along the *a*-axis. The structural complexity and tunability of ReSi_1.75_ originate from the ordered distribution of these vacancies in its crystal structure.^7,9^ The occurrence of the vacancies can be rationalized via the (18 – *n*) electron rule.^15,16^ For MoSi_2_ and ReSi_1.75_, *n* = 4, which means their optimum valence electron count is 14 per formula unit (f.u.). In MoSi_2_, Mo has 6 valence electrons, and the two Si have 2 × 4 = 8, so totally 14. Re has 7 valence electrons, so Si vacancies occur to maintain 14 (ReSi_1.75_□_0.25_: 7 + 1.75 × 4 + 0 × 0.25 = 14). These Si vacancies are ordered. When either Re or Al is substituted by another element with a different electron count, Si vacancies’ ordering can be altered. For instance, when we substitute Si with 0.1 Al per f.u., the expected composition is ReAl_0.100_Si_1.675_□_0.225_ to maintain 14 valence electrons, and it adopts an incommensurate structure, which is different from ReSi_1.75_□_0.25_.^9^

**Figure 1:**
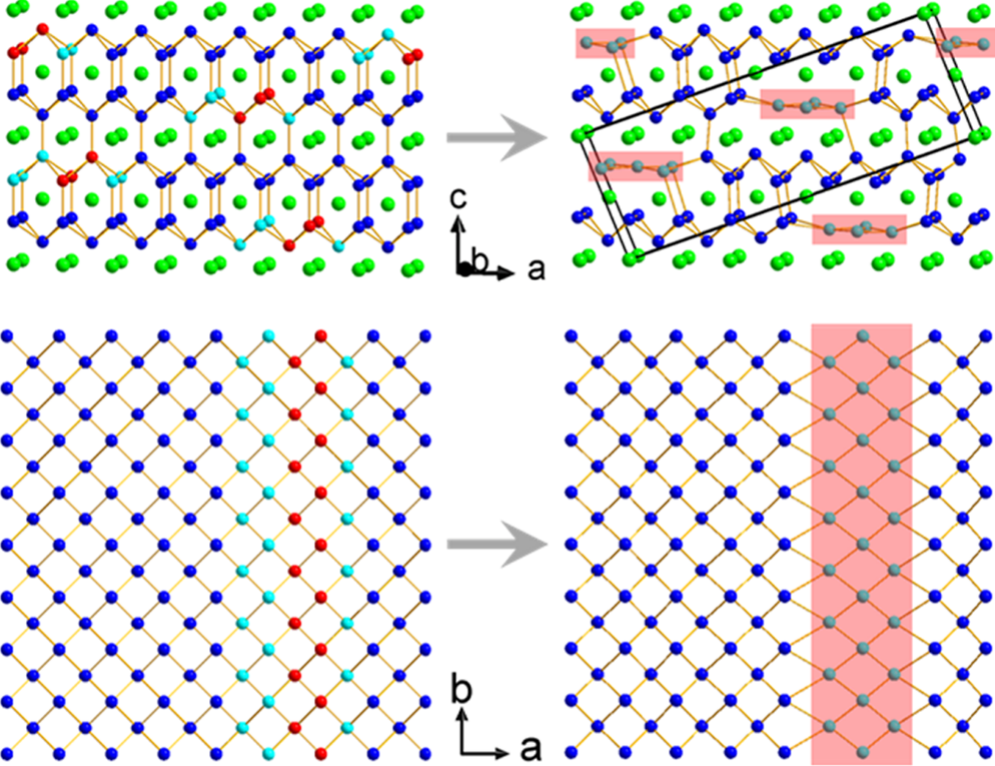
Si vacancies in ReSi_1.75_. The green atoms are Re, and the others are Si. From the MoSi_2_-type structure (left), the Si atoms in red are removed, and the neighboring Si atoms (in cyan) are displaced to compensate for the vacancies (highlighted in pink boxes).^9^

This report endeavors to thoroughly explore the structural variation and tunability of Al substitution in ReSi_1.75_□_0.25_. The substitution limit was previously determined to be 1.2 Al per f.u. (ReAl_1.2_Si_0.8_). Higher Al substitution causes the formation of other phases.^14^ For the whole substitution range, the Al-low end, ReSi_1.75_□_0.25_ and ReAl_0.100_Si_1.675_□_0.225_, and the Al-high end, ReAlSi and ReAl_1.2_Si_0.8_, have been studied and reported.^9,14^ We thus studied in this work the loading compositions between the two ends, ReAl_*x*_Si_1.75–0.75*x*_□_0.25–0.25*x*_ with *x* = 0.2–0.9. These loading compositions all satisfy the (18 – 4) electron rule. From single-crystal X-ray diffraction (XRD), besides the previously reported structures, we identified two new incommensurate structures which were successfully refined with (3 + 1)-dimensional superspace groups. First-principles calculations were performed to rationalize the stability of the new structures.

## Experimental Details

### Synthesis

Elemental Re (foil, 99.97%, Alfa Aesar), Si (rod, 99.999%, Alfa Aesar), and Al (wire, 99.999%, Alfa Aesar Puratronic) were mixed in a series of loading compositions, ReAl_*x*_Si_1.75–0.75*x*_□_0.25–0.25*x*_ (*x* = 0.2–0.9). The total mass of each mixture was 0.5000 ± 0.0005 g. The mixtures were arc melted in an argon atmosphere. Each sample was flipped and remelted three times to ensure a thorough reaction. The mass loss upon melting was less than 10 mg. The obtained silvery pellets were each split into two portions, one of which was sealed in an evacuated silica tube and annealed at 1000 °C for a week. The purpose of the annealing was to improve the crystallinity and check for possible phase transitions. Moreover, to test the reproducibility, the syntheses were repeated in several batches.

### X-ray Diffraction

Powder X-ray diffraction was performed using a Bruker D8 Discover diffractometer with Cu Kα radiation (λ_Kα1_ = 1.5405 Å, λ_Kα2_ = 1.5443 Å). The scan range of 2θ was 15–90° with a step size of 0.02°. Single-crystal diffraction was done for the majority of samples on a Rigaku Synergy-S diffractometer equipped with Mo Kα radiation (λ_Kα_ = 0.71073 Å). An Oxford Xcalibur E diffractometer equipped with Mo Kα radiation was used for a crystal picked from ReAl_0.20_Si_1.60_. The CrysAlis package was used for data integration, reduction, and absorption correction. The structural refinement, for both powder and single-crystal data, was done using Jana2006.^17^ As Si and Al cannot be differentiated, they are all treated as Si. This report focuses on the crystal structures and their variations upon Al substitution. The Al and Si atoms’ distribution, or the “coloring problem”,^18^ needs to be studied with neutron diffractions and deserves its own report.

### First-Principles DFT Calculations

Two commensurate approximant model structures (models 1 and 2) with the same composition, ReAl_0.2_Si_1.6_□_0.2_, were built to simulate the two new incommensurate structures found in this work. The details of the real and approximate structures are included in the Results and Discussion section. First-principles calculations were performed on these two approximant structures using the Vienna ab initio simulation package (VASP)^19–22^ running on “Mill” at the High-Performance Computing Center, Missouri University of Science and Technology.^23^ The Perdew–Burke–Ernzerhof (PBE)^24^ generalized gradient approximation was used to treat the electronic exchange and correlation. The projector wave augmented (PAW)^25^ pseudopotentials were used for Re, Si, and Al. The energy cutoff for the plane wave basis functions was 245.3 eV. The first Brillouin zone was sampled with a 3 × 13 × 5 Monkhorst mesh^26^ for model 1 and a 3 × 15 × 3 mesh for model 2. The model structures were optimized at the same fixed volume per f.u., 37.90 Å^3^, with the conjugate gradient algorithm.^27^ The energy convergence criterion is 5 × 10^–7^ eV/f.u. for the structural optimization and 5 × 10^–8^ eV/f.u. for the self-consistent electronic iterations. The crystal orbital Hamilton populations (COHPs)^28^ were calculated with the LOBSTER package.^29–32^

### Safety

There are no significant hazards or special safety concerns in the syntheses, crystallography, or computation performed in this work.

## Results and Discussion

### Single-Crystal XRD

Single crystals were picked from all samples in different batches for XRD. The results are consistent among batches. Within our loading composition range, ReAl_*x*_Si_1.75–0.75*x*_□_0.25–0.25*x*_, where *x* = 0.2–0.9, totally three distinct structures were observed. Among them, one is isotypical with the reported ReAlSi,^14^ and the other two are new structures. With these three structures, plus the previously reported ReSi_1.75_,^7,8^ ReAl_0.100_Si_1.675_□_0.225_,^9^ and ReAl_1.2_Si_0.8_,^14^ there are a total of six crystal structures identified at different Al substitution levels until now. They are numbered in this report as Structures I–VI in the order of increasing Al loading level: Structure I is the binary ReSi_1.75_;^7,8^ Structure II is ReAl_0.100_Si_1.675_□_0.225_;^9^ Structures III and IV are the two new structures identified in this work; and Structures V and VI are ReAlSi and ReAl_1.2_Si_0.8_,^14^ respectively.

Figure 2 lists the (*h*1*l*) precession images of the six structures constructed from single-crystal diffraction data. Al substitution “kaleidoscopes” the reciprocal lattices of ReSi_1.75_, resulting in six patterns with similar main reflections and different satellite reflections.

**Figure 2:**
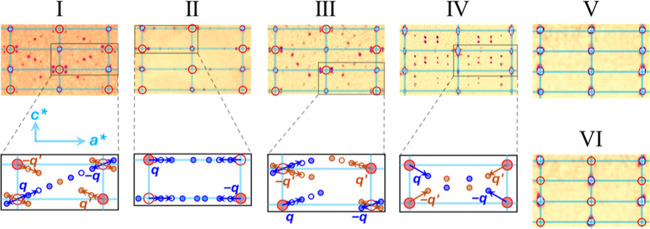
(*h*1*l*) precession images of Structures I through VI. The zoom-in sketches were drawn for Structures I through IV to show the satellites (blue) and ***q***-vectors (blue). The ***q*** ′ and satellites in brown in Structures I, III, and IV are related to the blue ***q*** and satellites through the twinning operation of rotating by 180° about c*.

The main reflections are the large spots in the precession images, marked as the big solid red dots in Figure 2 zoom-in sketches. For all structures, their main reflections can be indexed with the same reciprocal lattice, which is shown with the cyan grids in Figure 2 zoom-in sketches. Structures IV and V have main reflections at all the grid points, while Structures I, II, III, and VI have systematic absences shown with the big hollow red dots in Figure 2. The systematic absence is due to body-centering, which will be detailed below in crystal structures.

The satellite reflections are the small spots in the precession images, marked as the small solid blue and brown dots in Figure 2 zoom-in sketches. Structures V and VI have only main reflections and no satellites. Structures I through IV all have satellites, and they are patterned differently. The satellites’ reflections can be indexed with reciprocal wave vectors, ***q*** = σ_1_ a* + σ_2_ b* + σ_3_ c*, or simply (σ_1_, σ_2_, σ_3_), where a*, *b* *, and c* are reciprocal lattice vectors for the main reflections. ***q*** is usually chosen as the vector pointing from a main reflection to its nearest/strongest satellite. The ***q***-vectors are shown in Figure 2 zoom-in sketches and quantitatively compared in Table 2 for Structures I through IV. Structure I, the binary ReSi_1.75_, has a ***q*** exactly 1/8 of a* + c*, or ***q***_I_ = (1/8, 0, 1/8). σ_1_ = σ_3_ = 1/8, a simple rational number, indicates Structure I is a commensurate structure and can be depicted with either a supercell (*Cm*)^6–8^ or a (3 + 1)-dimensional superspace group, *X*2/*m*(α0γ).^9^ Structure II’s ***q*** is horizontal along a* and slightly shorter than 1/8a*, ***q***_II_ = (0.1201, 0, 0). Structures III and IV are new structures. The precession image of Structure III is similar to Structure I, but III’s ***q***_III_ = (0.1106, 0, 0.0758) is shorter and less tilted from a*. Structure IV’s ***q***_IV_ = (0.1914, 0, −0.3021) is much longer and more tilted from a* than all the other structures’ ***q*** vectors. Moreover, Structures I, II, and III have systematic absence in both main (hollow big red dots in Figure 2) and satellite reflections (hollow small blue and brown dots in Figure 2), while Structure IV has no systematic absence at all. For Structures II, III, and IV, the σ_1_ and/or σ_3_ in ***q*** are not simple rational numbers, so they are incommensurate structures and can only be depicted with (3 + 1)-dimensional superspace groups. Structure II was reported in superspace group *X*2*mm*(α00)*s*0*s*.^9^ Structures III and IV are detailed below in the discussion of crystal structures.

Structures III and IV were solved and refined from single-crystal diffraction data, and the refinement details can be found in Table 1. More detailed information, including atomic positions and modulation waves, can be found in Supporting Information and the CIF files submitted along with it. All six structures are shown and compared in Figure 3. Structure VI, ReAl_1.2_Si_0.8_, is isotypic with MoSi_2_, body-centered tetragonal (*I*4/*mmm*) with Al and Si statistically sharing the same sites and no vacancy.^14,33^ The body-centering causes the systematic absence of the main reflections in its precession image in Figure 2. Structure V, ReAlSi, is almost the same as Structure VI, tetragonal and no Si/Al vacancy, except that, instead of being statistically mixed, Al and Si atoms are segregated into alternating (001) layers, as proved by neutron diffraction.^14^ The Si/Al ordering eliminates the body-centering, making the unit cell primitive tetragonal (*P*4/*nmm*), and thus, there is no systematic absence of main reflections in Structure V’s precession image in Figure 2.

**Table 1:** Single-Crystal XRD Data Collection and Refinement Information of the Two New Incommensurate Structures

	Structure III	Structure IV
refined composition	Re(Al,Si)_1.7788_	Re(Al,Si)_1.8086_
crystal system, space group	monoclinic, *X*2/*m*(α0γ)00	monoclinic, *P*2_1_/*m*(α0γ)00
***q***-vectors	0.1106 ***a*** * + 0.0758 ***c*** *	0.1914 ***a*** * – 0.3021 ***c*** *
*a*, *b*, *c* (Å)	3.131(4), 3.148(3), 7.733(3)	3.1511(2), 3.1538(3), 7.7648(7)
β (deg)	89.95(6)	90.024(7)
*V* (Å^3^)	76.22(13)	77.17(1)
*Z*	2	2
crystal size (mm)	0.07 × 0.05 × 0.04	0.05 × 0.05 × 0.04
no. of measured, independent, and observed [*I* > 3σ(*I*)] reflections	2965, 857, 413	19,143, 2335, 1803
*R*_int_	0.068	0.037
*R*[*F*^2^ > 2σ(*F* ^2^)], *wR*(*F* ^2^), *S*	0.048, 0.057, 1.51	0.037, 0.064, 2.85
main *R*[*F* ^2^ > 2σ(*F* ^2^)], *wR*(*F* ^2^)	0.037, 0.045	0.030, 0.054
first *R*[*F* ^2^ > 2σ(*F* ^2^)], *wR*(*F* ^2^)	0.077, 0.104	0.065, 0.075
second *R*[*F* ^2^ > 2σ(*F* ^2^)], *wR*(*F* ^2^)	0.087, 0.154	0.071, 0.088
third *R*[*F* ^2^ > 2σ(*F* ^2^)], *wR*(*F* ^2^)	0.102, 0.151	
fourth *R*[*F* ^2^ > 2σ(*F* ^2^)], *wR*(*F* ^2^)	0.132, 0.231	
no. of parameters/restraints	61/0	93/0
Δρ_max_, Δρ_min_ (e Å^–3^)^a^	3.53, −6.86	7.40, −5.02

a The positions and cause of large peaks/holes are discussed in Supporting Information.

**Figure 3:**
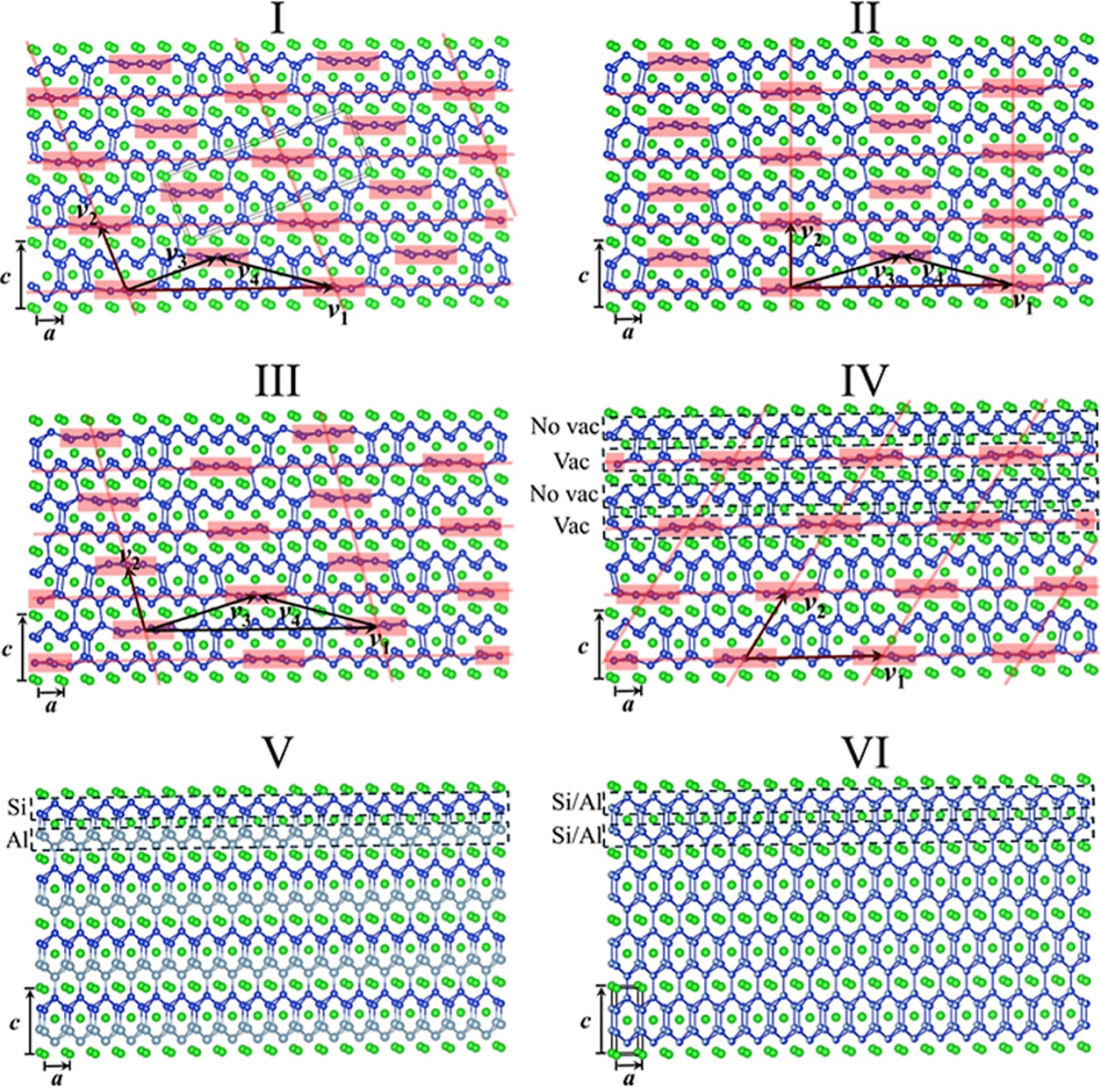
Crystal structures of Structures I through VI. Atom color code: Re—green; Si—dark blue; and Al—light blue.

Structures I through IV have both main and satellite reflections. From the main reflections, we determined their “basic structures”. The basic structures of Structures I, II, and III are isotypic with Structure VI (*I*4/*mmm*), while Structure IV’s basic structure is isotypic with Structure V (*P*4/*nmm*). Including the satellite reflections and applying (3 + 1)-dimensional superspace groups, the “full structures” can be solved for I through IV, whose superspace groups are *X*2/*m*(α0γ), *X*2*mm*(α00)*s*0*s*, *X*2/*m*(α0γ), and *P*2_1_/*m*(α0γ), respectively. As shown in Figure 3, they all feature Si/Al vacancies similar to those described in Introduction and Figure 1, indicated by the “flat” Si/Al atoms highlighted with pink boxes. In all four structures, the vacancies are not randomly distributed but are ordered and form their own lattice (pink grid in Figure 3) defined by basis vectors ***v***_1_ and ***v***_2_ as shown in Figure 3. ***v***_1_ and ***v***_2_ can be quantified with the ***q***-vector and the basic structure’s basis vectors, ***a*** and ***c***. As shown in Table 2, the ***q***-vectors all have the general form of (σ_1_, 0, σ_3_), with σ_3_ = 0 for Structure II. For all four structures, $v_{1}=\frac{1}{\sigma_{1}}a$ and $v_{2}=-\frac{\sigma_{3}}{\sigma_{1}}a+c$ . For Structures I, II, and III, the vacancy lattices are “centered” with the centering vector $v_{3}=\frac{1}{2}\left[v_{1}+v_{2}\right]$ . By comparison, Structure IV’s vacancy lattice has no ***v***_3_. This is because I, II, and III have vacancies in every single Si/Al(001) slab, while IV has vacancies in every second Si/Al(001) slab (shown with alternating “Vac” and “No Vac” in Figure 3). The vacancy lattice vectors, ***v***_1_, ***v***_2_, ***v***_3_, and $v_{4}=\frac{1}{2}\left[-v_{1}+v_{2}\right]$ , are listed in Table 2, too.

**Table 2:** ***q***-Vectors and Compositions of Structures I through IV

Structure	q-vector	composition	***v***_1_	***v***_2_	***v***_3_	***v***_4_
I^a^	(1/8, 0, 1/8)	ReSi_1.75_□_0.25_	8 ***a***	–***a*** + ***c***	3.5 ***a*** + 0.5 ***c***	–4.5 ***a*** + 0.5 ***c***
II^b^	(0.1201, 0, 0)	Re(Al,Si)_1.7598_□_0.2402_	8.326 ***a***	***C***	4.163 ***a*** + 0.5 ***c***	–4.163 ***a*** + 0.5 ***c***
III	(0.1106, 0, 0.0758)	Re(Al,Si)_1.7788_□_0.2212_	9.042 ***a***	–0.685 ***a*** + ***c***	4.179 ***a*** + 0.5 ***c***	–4.864 ***a*** + 0.5 ***c***
IV	(0.1914, 0, −0.3021)	Re(Al,Si)_1.8086_□_0.1914_	5.225 ***a***	1.578 ***a*** + ***c***		

a Reported in ref 7.

b Reported in ref 9.

$v_{1}=\frac{1}{\sigma_{1}}a$ indicates that vacancies occur every $\frac{1}{\sigma_{1}}$ basic unit cell along the *a*-axis in a Si/Al(001) slab. This allows us to formulate the compositions of Structures I, II, and III (with vacancies in every single Si/Al(001) slab) as ${Re\left(Al,Si\right)}_{2\left(1-\sigma_{1}\right)}{□}_{2\sigma_{1}}$ and the composition of Structure IV (with vacancies in every second Si/Al(001) slab) as Re­(Al,Si)_2−σ1_□_σ1_. Using the experimental σ_1_ values, the compositions for Structures I through IV are also formulated in Table 2. The number of vacancies per formula unit monotonously decreases from I to IV.

The occurrence and ordering of Si/Al vacancies has been rationalized in our previous report.^9^ There are three factors that determine how vacancies occur and distribute: (1) Si/Al vacancies occur so that the valence electron count does not exceed 14 per f.u. This is why Structures V (ReAlSi, exactly 14 e^–^) and VI (ReAl_1.2_Si_0.8_, less than 14 e^–^)^14^ have no vacancies. (2) The Si/Al vacancies occur, as described in Introduction and Figure 1, by removing two neighboring Si/Al rows along the ***b***-axis, which allows the Si/Al atoms nearby to displace and most effectively fill the voids. This is how vacancies occur in Structures I and II as well as in the two new structures, III and IV. (3) It is energetically favorable to have vacancies stay as far away as possible from their neighboring vacancies in the same and the immediately adjacent Si/Al(001) slabs, causing their ordered distribution rather than random arrangements. In other words, the Si/Al vacancies are ordered to maximize the length of ***v***_1_ (| ***v***_1_|), the distance between neighboring vacancies in the same Si/Al(001) slab, and also to maximize the lengths of $v_{3}=\frac{1}{2}\left[v_{1}+v_{2}\right]$ and $v_{4}=\frac{1}{2}\left[-v_{1}+v_{2}\right]$ (| ***v***_3_| and | ***v***_4_|), the distances between neighboring vacancies from two adjacent Si/Al(001) slabs.

To maximize | ***v***_1_|, the vacancies should be evenly distributed and equidistantly spaced in all Si/Al(001) slabs, not just some of the slabs. This is the case in Structures I, II, and III but not the case in Structure IV, whose vacancies are crowded into every second Si/Al(001) slab, while the other slabs have no vacancies. Structure IV’s | ***v***_1_| could be doubly longer if its vacancies were dispersed evenly into every single Si/Al(001) slab like in the other three structures. The reason why Structure IV squeezes its vacancies into every second Si/Al(001) slab is discussed in “Rationalizing the Vacancy Distribution in Structure IV”.

To maximize | ***v***_3_| and | ***v***_4_|, the vacancies of two neighboring Si/Al(001) slabs need to be perfectly “staggered”, i.e., $\left|v_{3}\right|=\left|v_{4}\right|=\frac{1}{2}\left|\pm v_{1}+c\right|$ . This is exactly the case in Structure II. For Structures I and III, the vacancies are not perfectly “staggered”, and | ***v***_3_| is slightly shorter than | ***v***_4_| as $\left|v_{3}\right|=\frac{1}{2}\left|\left(1-\sigma_{3}\right)v_{1}+c\right|$ and $\left|v_{4}\right|=\frac{1}{2}\left|-\left(1+\sigma_{3}\right)v_{1}+c\right|$ . However, our previous report^9^ revealed that this deviation from perfect staggering only causes a few meV difference per formula unit in energy, which can be easily overcome at room temperature.

### Powder XRD

Powder XRD was done to identify the structures present in all samples, which are listed in Table 3. The powder diffraction patterns with LeBail refinement and detailed discussion on phase analyses are included in the Supporting Information.

**Table 3:** Structures Identified with Powder XRD in All Samples^a^

sample load. comp.	as-cast	annealed
ReAl_0.2_Si_1.6_	**III** + ReSi + Re_2_Si	**III** + V + Re_2_Si
ReAl_0.3_Si_1.525_	**III** + **IV**	**III** + V
ReAl_0.4_Si_1.45_	**III** + **IV**	**III** + V
ReAl_0.5_Si_1.375_	**IV** + ReSi	**III** + **V**
ReAl_0.6_Si_1.3_	**IV** + V + ReSi	**III + V**
ReAl_0.7_Si_1.225_	**III** + **IV** + V + ReSi	**III + V**
ReAl_0.8_Si_1.15_	III + **IV** + **V** + ReSi	**III + V**
ReAl_0.9_Si_1.075_	V	III + **V**

a The bold font indicates the major phases.

As shown in Table 3, in addition to the target ReAl_*x*_Si_1.75–0.75*x*_ phases, ReSi- and Re_2_Si-type structures were also observed as minor phases in our samples. With increasing Al substitution, the main phase varies from Structure III to Structure IV and then to Structure V in the as-cast samples. Upon annealing at 1000 °C, all samples transform into mixtures of Structures III and V. Structure IV was found only in the as-cast samples. This indicates that Structure IV is a metastable phase, and it transforms into Structures III and V during annealing.

### Rationalizing the Vacancy Distribution in Structure IV

As mentioned above, Structure IV is different from Structures I, II, and III in the ordering of Si/Al vacancies. While I, II, and III have their vacancies evenly distributed in all Si/Al(001) slabs, Structure IV crowds all vacancies in every second Si/Al(001) slab and leaves the other slabs with no vacancies. To rationalize this uniqueness of Structure IV, we constructed two model structures with the same composition, ReAl_0.2_Si_1.6_□_0.2_, which satisfies 14 e^–^ per f.u., and did first-principles calculations upon them. Figure 4 shows the two model structures. The first model structure (model 1) is a commensurate approximant for Structure IV. It has a ***q***-vector of (0.2, 0, −0.3), simulating Structure IV’s experimental ***q***-vector of (0.1914, 0, −0.3021). Like Structure IV, model 1 crowds all vacancies in every second Si/Al(001) slab. The distance between neighboring vacancies within the same slab is | ***v***_1_| = |5 ***a*** |. Also, Al atoms are all in the vacancy slabs, and the no-vacancy slabs are pure Si slabs. The second model structure (model 2) is a hypothetical structure with a ***q***-vector of (0.1, 0, 0). In model 2, vacancies are distributed in every single Si/Al(001) slab and thus are dispersed farther apart than in model 1. Model 2’s distances between neighboring vacancies are | ***v***_1_| = |10 ***a*** | within the same slab, and $\left|v_{3}\right|=\left|5a+\frac{1}{2}c\right|$ and $\left|v_{4}\right|=\left|-5a+\frac{1}{2}c\right|$ between neighboring vacancies from adjacent slabs, all of which are longer than the |5 ***a*** | in model 1. A comparison between these two model structures will thus shed light on why Structure IV crowds its Si/Al vacancies in every second Si/Al(001) slab. More details about model 1 and model 2, such as space groups, lattice parameters, and atomic positions, can be found in the Supporting Information.

**Figure 4:**
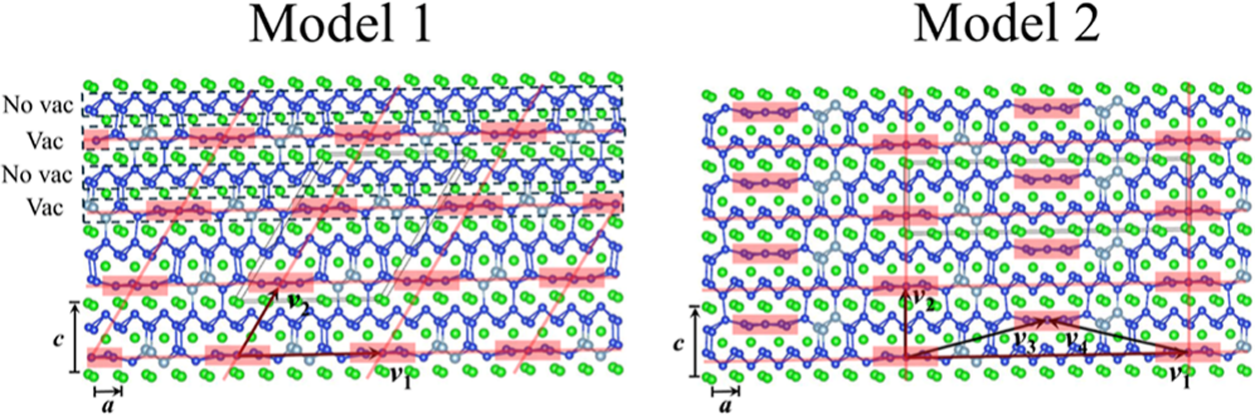
Model structures for first-principles computations. Atom color code: Re—green; Si—dark blue; and Al—light blue.

Both model structures were first optimized for unit cell aspect ratios and atomic positions to minimize their total energies, which are compared in Table 4. Model 1 is higher (less negative) than model 2 in total energy, which means that model 1 is energetically less stable than model 2. This is consistent with the observation that Structure IV, simulated as model 1, is metastable only and transforms into other structures upon annealing. Therefore, crowding all vacancies in every second Si/Al(001) slab, such as Structure IV and model 1, is energetically unfavorable. Dispersing vacancies evenly in all Si/Al(001) slabs, like in Structures I, II, and III and model 2, is indeed energetically favorable, like we argued in our previous report.^9^

**Table 4:** Computational Comparison between Model 1 and Model 2

	model 1		model 2
total energy per cell (eV)	–458.0788		–458.1107
Re–Si ICOHP per cell (eV)	–358.5156		–357.8571
no. Re–Si contacts per cell	154	100 (no vac)	154
		54 (vac)	
average Re–Si ICOHP per bond (eV)	–2.3280	–2.3611 (no vac)	–2.3237
		–2.2667 (vac)	
average Re–Si distance (Å)	2.5403	2.5175 (no vac)	2.5409
		2.5826 (vac)	

The remaining question is, even though it is less stable, why does Structure IV still occur in some arc-melted samples? Clues can be found in Structure V, ReAlSi, which, even though lacking vacancies, also has two different kinds of Si/Al(001) slabs, pure Si slabs and pure Al slabs, alternating. It was rationalized previously^14^ that the Si and Al atoms segregate into alternating (001) slabs in Structure V because pure Si(001) slabs, compared to Si/Al mixed slabs, allow shorter Re–Si distances and stronger Re–Si bonding, which are the strongest bonds (compared to Re–Al, Si–Si, and Si–Al) and contribute the most in stabilizing the compound. Likewise, we suspect that Structure IV also crowds all vacancies and Al atoms into the vacancy-slabs, making the no-vacancy-slabs pure Si slabs, which afford stronger Re–Si bonding just like in Structure V. This conjecture is supported by our observation in single-crystal XRD that Structures IV and V frequently epitaxially intergrow into each other (Figure S5 in Supporting Information), which implicates that they very possibly share the same structural moieties (i.e., pure Si slabs) and stabilization mechanism.

To test this conjecture computationally, we investigated the Re–Si bonding in model 1, especially in its no-vacancy pure Si(001) slabs, and compared it with model 2. The comparison was made on the Re–Si bond lengths and integrated crystal orbital Hamilton populations (ICOHPs, a semiquantitative evaluation of bond energy) in Table 4. The two model structures both have 154 Re–Si bonds per unit cell. Their average Re–Si bond lengths are virtually equal, 2.5403 and 2.5409 Å, respectively. However, model 1 boasts stronger Re–Si bonding overall because its Re–Si ICOHP per unit cell (−358.5156 eV) is more negative than model 2 (−357.8571 eV).

Also, compared to model 2, model 1’s no-vacancy pure Si(001) slabs have shorter Re–Si average distance and more negative average Re–Si ICOHP, both indicating stronger bonding, while its vacancy Si/Al(001) slabs have longer Re–Si average distance and less negative average Re–Si ICOHP, both meaning weaker bonding.

So model 1 features stronger Re–Si bonding than model 2 overall, thanks to the shorter and stronger Re–Si bonds in the no-vacancy pure Si(001) slabs. Our conjecture about structure IV is thus verified: even though crowding vacancies in every second Si/Al(001) slab is energetically unfavorable, the no-vacancy pure Si slabs afford shorter Re–Si distances and stronger Re–Si bonding, which lend the metastable Structure IV achievability in arc melting syntheses.

Such crowding vacancies in exchange for stronger Re–Si bonding is feasible only when the number of vacancies is sufficiently low. Otherwise, crowding vacancies into every second Si/Al(001) slab would cause too short | ***v***_1_|. For instance, with the binary ReSi_1.75_□_0.25_, if all vacancies were crowded into every other slab, | ***v***_1_| would be only |4 ***a*** |, much shorter than the |5.225 ***a*** | in Structure IV. Too short | ***v***_1_| would compromise any benefit from strengthening Re–Si bonding, making it infeasible even as a metastable phase. As shown in Table 2, Structure IV has the fewest vacancies among I through IV. The other three structures all have over 0.22 vacancies, while Structure IV’s vacancy is below 0.2 per formula unit. So, vacancy crowding can only be achieved when the number of vacancies is below 0.2 per formula unit.

## Conclusions

Two new incommensurate structures (III and IV) were identified and successfully solved in the series of Al-substituted rhenium silicides, ReAl_*x*_Si_1.75–0.75*x*_□_0.25–0.25*x*_. With the previously reported ones, there are a total of six crystal structures found in this series, which are all derivatives of the MoSi_2_-type structure. Among them, two structures (V and VI) have no vacancies, while the other four (I through IV) bear similar Si/Al vacancies that are ordered differently. Several principles dictate the occurrence and ordering of Si/Al vacancies. Due to the (18 – 4) electron rule, the higher the Al-substitution level is, the fewer vacancies there are. It is energetically favorable to space the vacancies far away from each other in the same and the adjacent Si/Al(001) slabs, which is achieved by equidistantly distributing vacancies in every single Si/Al(001) slab and staggering vacancies between adjacent slabs. Structures satisfying these principles (I, II, and III) are thermodynamically stable and do not experience phase change upon annealing, as found in this and previous reports.^9,14^ Meanwhile, pure Si no-vacancy (001) slabs afford stronger Re–Si bonding and are thus also energetically desirable, with an unfavorable price of crowding vacancies in every second Si/Al(001) slab, and can only be achieved when the number of vacancies is below 0.2 per formula unit, resulting in the only metastable structure (IV) that transformed into other structures upon annealing.

With this knowledge, we now have a comprehensive understanding of the composition–structure relationship in ReAl_*x*_Si_1.75–0.75*x*_□_0.25–0.25*x*_. The remaining questions include the exact number of Al atoms and how they are distributed, i.e., the “coloring problem”,^18^ which entails further studies with both experimental, such as neutron diffraction, and theoretical techniques, such as first-principles calculations. Furthermore, the physical properties, such as thermoelectricity and their relationship with the crystal structures, can also be investigated, especially for the two new incommensurate structures.

## Supplementary Material







## Notes

More details about single-crystal refinement of Structures III, IV, and V; discussion on the residual electron density peaks/holes; phase identification and LeBail refinement on powder XRD patterns; epitaxial intergrowth between Structures IV and V; and details about computational model structures (PDF)
X-ray crystallographic data for Re-Si-Al Structure III [CCDC 2434810 does not have the complete information due to its superspace group] (CIF)
X-ray crystallographic data for Re-Si-Al Structure IV [CCDC 2434811 does not have the complete information due to its superspace group] (CIF)
