# Supplementary material for: Kaleidoscopic Incommensurate Structures of Aluminum-Substituted Rhenium Silicides
Source: Inorg Chem. 2025 Jun 23;64(26):13163–9. doi: 10.1021/acs.inorgchem.5c01400 (PMC12239066; doi:10.1021/acs.inorgchem.5c01400)
Supplement: Supplementary file 1 [file ic5c01400_si_001.pdf]

Supporting Information

Kaleidoscopic Incommensurate Structures of Aluminum-Substituted Rhenium Silicides

Victoria Decocq† and Fei Wang*,†

†Department of Chemistry and Biochemistry, Missouri State University, Springfield, MO 65897, United States

*Corresponding author, email: feiwang@missouristate.edu

1. Atomic Parameters and Positional Modulation Wave Parameters ……………………….…S2

2. Residual Electron Density Peaks/Holes in Single Crystal Refinement ……………………S4

3. LeBail Refinement on Powder XRD Patterns ………….……………………………………...S6

4. Details on Powder XRD Refinement ……………………………………………………..S8

5. Lattice Parameters from LeBail Refinement ………………………………………………….S9

6. Epitaxial Intergrowth between Structure IV and V ………………………………………S11

7. Details of Computational Model Structures ……………..………………………………S12

Atomic Parameters and Positional Modulation Wave Parameters

Table S1. Fractional atomic coordinates and isotropic or equivalent isotropic displacement parameters (Å^2) of Structure III and IV.

Structure III	x	y	z	U_{iso}*/U_{eq}
Re1	0.5	0.5	0	0.0082 (3)
Si1	0.7566 (19)	0	0.1789 (5)	0.0116 (14)
Structure IV	x	y	z	U_{iso}*/U_{eq}
Re1	0.74919 (6)	0.25	0.74217 (3)	0.01054 (8)
Si1	0.2539 (6)	0.75	0.5934 (2)	0.0037 (3)
Si2	0.7637 (8)	0.25	0.0491 (3)	−0.0098 (9)

Table S2. Atomic displacement parameters (Å^2) of Structure III and IV.

Structure III	U^{11}	U^{22}	U^{33}	U^{12}	U^{13}	U^{23}
Re1	0.0073 (5)	0.0083 (5)	0.0091 (5)	0	−0.0006 (9)	0
Si1	0.011 (3)	0.017 (2)	0.0071 (19)	0	0.001 (2)	0
Structure IV	U^{11}	U^{22}	U^{33}	U^{12}	U^{13}	U^{23}
Re1	0.01009 (14)	0.01121 (16)	0.01033 (14)	0	−0.00027 (12)	0
Si1	0.0055 (5)	0.0027 (5)	0.0029 (7)	0	0.0010 (8)	0
Si2	−0.040 (2)	0.0160 (9)	−0.0049 (11)	0	−0.0007 (11)	0

The atomic positions listed in Table S1 are the average positions in basic structures. The actual atomic positions are these average positions plus a displacement, $u(v)$:

$$u(v) = \sum_n u_n^c \cos(2\pi n v) + u_n^s \sin(2\pi n v)$$

where $v = \boldsymbol{q} \cdot \boldsymbol{t}$, the dot product between $\boldsymbol{q}$-vector and a lattice translation vector, $\boldsymbol{t}$. The u_n^c and u_n^s parameters are listed in Table S3.

Table S3. Atomic positional modulation wave parameters for Structure III and IV.

Structure III	n	$u^c_{n,x}$	$u^c_{n,y}$	$u^c_{n,z}$	$u^s_{n,x}$	$u^s_{n,y}$	$u^s_{n,z}$
Re1	1	0	0	0	0.0002(4)	0	0.0111(2)
	2	0	0	0	–0.0131(4)	0	0.00323(13)
	3	0	0	0	0.0052(4)	0	0.00681(17)
	4	0	0	0	–0.0051(4)	0	–0.00406(15)
Si1	1	0.224(2)	0	0.0017(7)	0.000(3)	0	–0.0398(7)
	2	0.002(2)	0	–0.0213(6)	–0.050(3)	0	0.0058(8)
	3	0.007(2)	0	0.0015(8)	–0.003(2)	0	0.0127(7)
	4	–0.001(3)	0	0.0077(10)	–0.028(2)	0	–0.0012(9)
Structure IV	n	$u^c_{n,x}$	$u^c_{n,y}$	$u^c_{n,z}$	$u^s_{n,x}$	$u^s_{n,y}$	$u^s_{n,z}$
Re1	1	0.00748(12)	0	–0.00029(4)	–0.01300(14)	0	–0.00457(6)
	2	0.00079(10)	0	0.00645(7)	0.00148(9)	0	0.00815(8)
Si1	1	0.0044(6)	0	–0.0051(3)	0.0014(7)	0	–0.0105(3)
	2	–0.0006(7)	0	0.0006(3)	–0.0004(7)	0	0.0003(3)
Si2	1	0.0025(12)	0	0.0493(4)	–0.1031(18)	0	–0.0083(5)
	2	–0.0108(16)	0	–0.0132(6)	–0.0404(16)	0	0.0026(6)

For more details, the cif files for aperiodic Structure III and IV can be found along with the Supporting Information. To visualize the structures, we need to open the cif files with Jana2006 or Jana2020 (free software) and generate 3-D approximant structure. The approximant structures can be written into temporary cif files and visualized using Mercury, Diamond, or VESTA.

Residual Electron Density Peaks/Holes in Single Crystal Refinement

The largest residual electron density peaks and holes in single crystal refinement are relatively large, 3.52/−6.86 e/Å^3 for Structure III and 7.40/−5.02 e/Å^3 for Structure IV. The positions of these peaks and holes in the unit cells were examined and shown in Figure S1. In both structures, the largest peaks are located next to the Re atoms and the deepest holes are around or even coincide with the Si/Al atoms in the (001) slabs bearing vacancies.

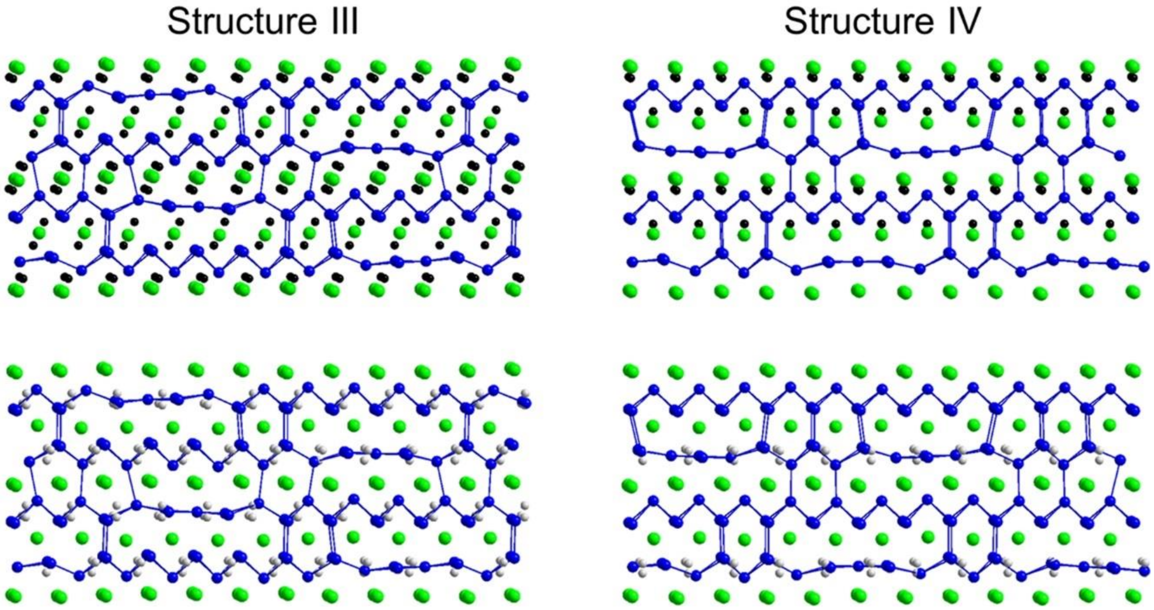

Figure S1. The residual electron density peaks (dark) and holes (gray) in Structure III and IV. Atom color code: Re – green; Si – dark blue.

Several strategies were adopted in attempt to reduce the peaks and holes, including adding new twinning domains, adding new atoms with partial occupancies, adding more modulation waves to atomic displacement parameters (ADP), and adding a second structure (like Structure V) as an epitaxial minor phase. However, none of these attempts affords a significant improvement in refinement. For instance, adding more modulation waves to Si's (ADP) lowered Structure III's deepest residual hole from −6.86 e/Å^3 to −4.29 e/Å^3 without noticeably changing the R values (from 0.048 to 0.047). The price of this minor improvement is increasing the number of refinement parameters from 61 to 85.

The cause of the relatively large residual peaks/holes can be attributed to twinning or intergrowth between different structures. For instance, Structure IV was found intergrowing epitaxially with Structure V (see "Epitaxial Intergrowth between Structure IV and V" in this Supporting Information). The mismatch in atomic positions between the two structures could cause the residual peaks and holes. Similar situation could also occur between Structure III and II, which causes the peaks and holes in Structure III's refinement.

To eliminate the large residual electron density peaks/holes, pure phase samples are needed. This can be achieved by fine tuning the loading composition in syntheses and annealing at different temperatures. In pure phase samples, without twinning or intergrowth, the refinement is expected to give lower peaks and holes. This will be tried in our future study.

LeBail Refinement on Powder XRD Patterns

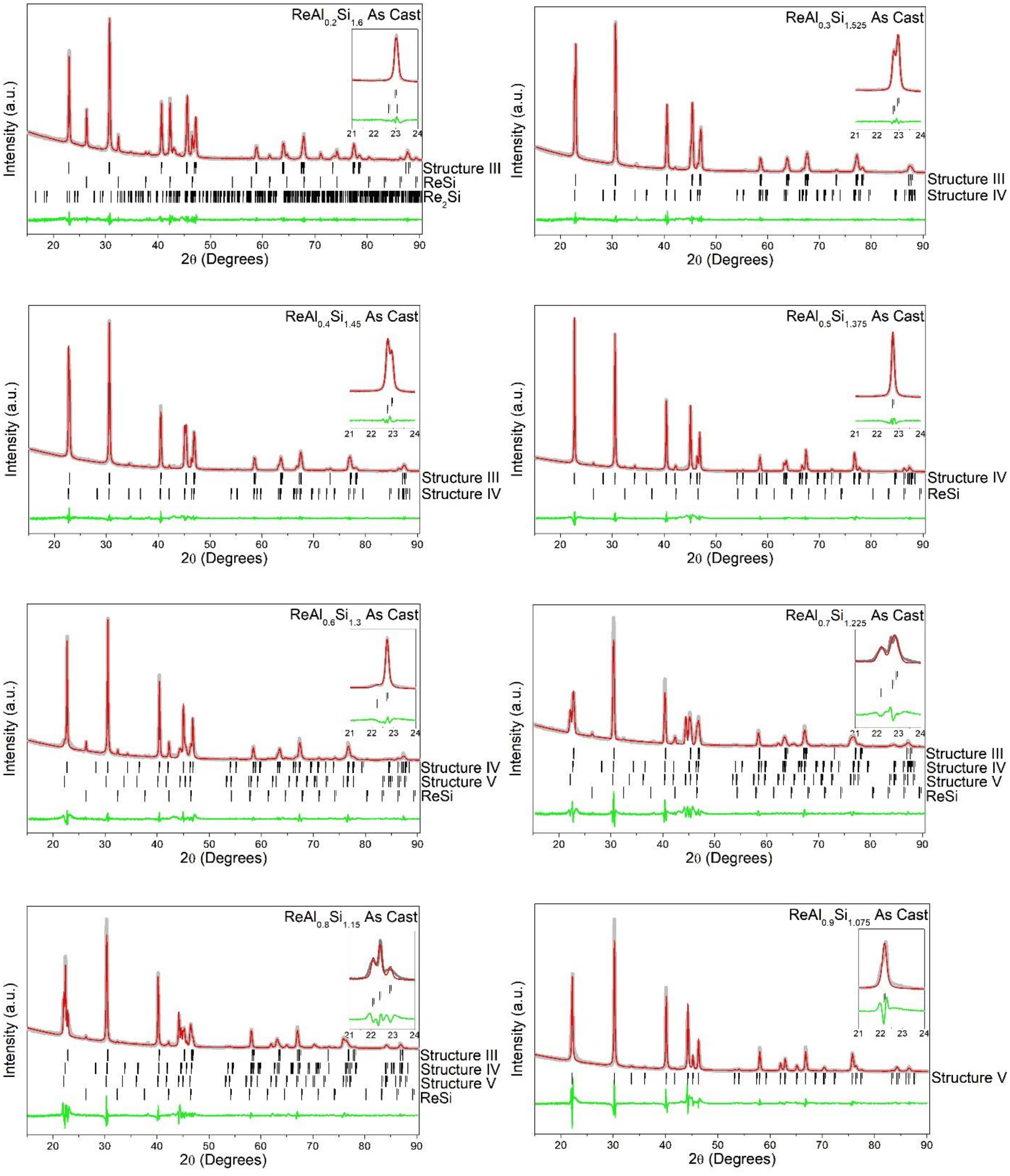

Figure S2. The powder XRD patterns of the as-cast samples. The insets show the (002) peaks of the $MoSi_2$-type basic structures.

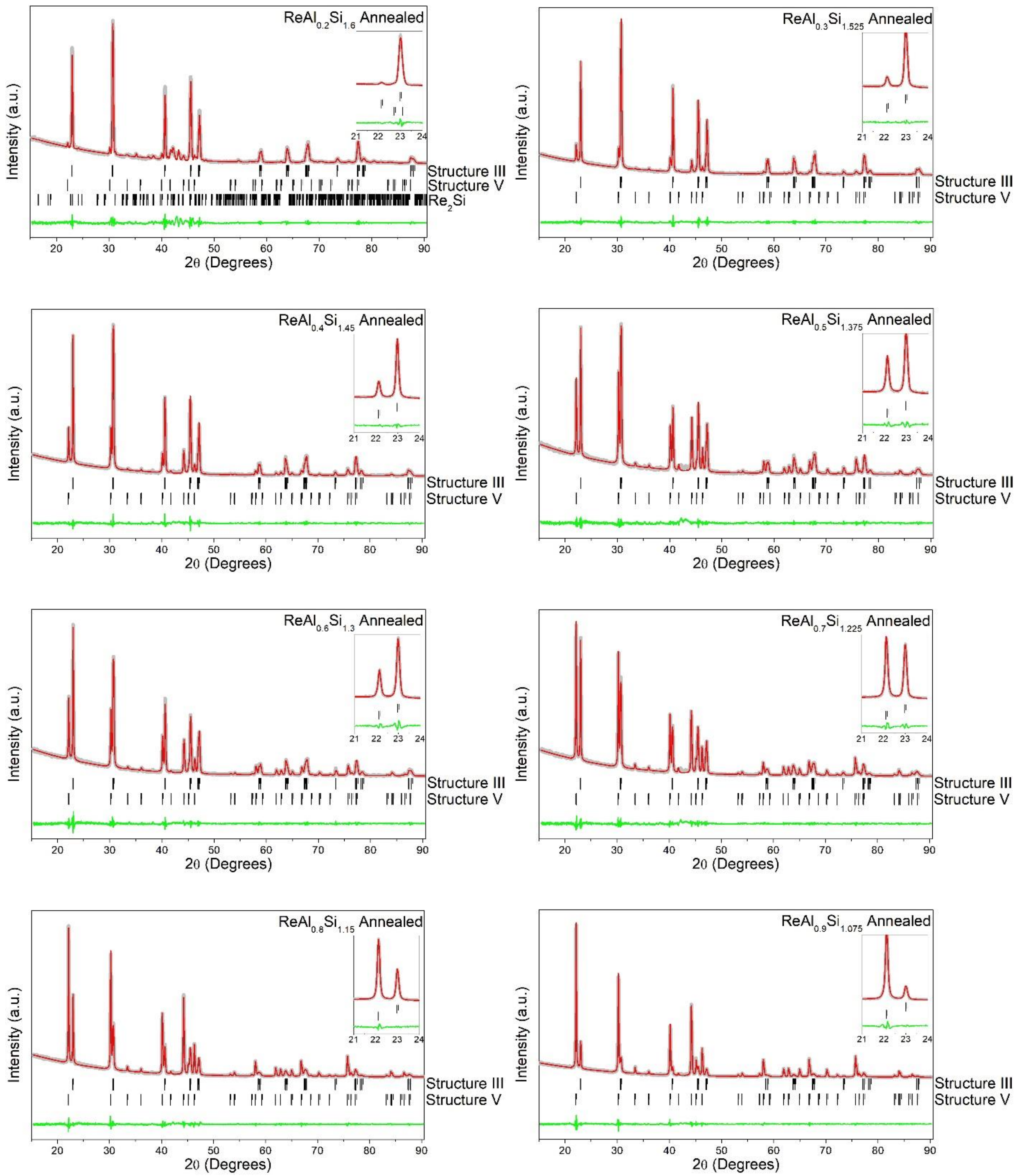

Figure S3. The powder XRD patterns of the annealed samples. The insets show the (002) peaks of the $MoSi_2$-type basic structures.

Details on Powder XRD Refinement

Powder XRD patterns were used to identify the presence of Structure III, IV, and V (the only structures found in single crystal XRD) in all $ReAl_xSi_{1.75-0.75x}\square_{0.25-0.25x}$ (x = 0.2 – 0.9) samples. Even though their satellite reflections are distinct, satellite peaks are too weak to be visible in powder diffraction patterns so they cannot be relied on to identify the three structures. Instead, we used the main reflection peaks only, from which the basic structure lattice parameters can be obtained through LeBail refinement. The challenge is that the three structures have very close basic structure lattice parameters so their main reflection peaks often overlap or even coincide. When two or three structures coexist, it is difficult to deconvolute overlapping/coinciding peaks and LeBail refined lattice parameters can be inaccurate. Fortunately, the *c* parameters of Structure III, IV, and V are sufficiently different so that the (002) peaks located between 2θ = 22° and 23° (The insets in Figure S2 and S3) is reliable in discerning how many structures are present. The refined basic structure lattice parameters are plotted (Figure S4) and tabulated (Table S4 and S5) below.

The annealed samples' diffraction patterns are simple. They can all be refined with Structure III and Structure V. Only the two samples with loading compositions of $ReAl_{0.2}Si_{1.6}$ and $ReAl_{0.5}Si_{1.375}$ have Re_2Si as the minor phase (Figure S3). The refined basic structure lattice parameters for both Structure III and V are consistent across all the series of samples and also close to the lattice parameters obtained from single crystal refinement (Figure S4). From the (002) peaks, it is evident that Structure V's (002) peak at ~22° grows higher and higher while Structure III's (002) peak at ~23° gets lower and lower with increasing Al loading. In conclusion, for $ReAl_xSi_{1.75-0.75x}\square_{0.25-0.25x}$ (x = 0.2 – 0.9) samples, Structure III and V are the two thermodynamically stable phases at the annealing temperature, 1000 °C. Vary Al loading level alters the ratio between Structure III and V in the annealed samples.

The as-cast samples are more complicated (Figure S2). Besides Structure III and V, there are also Structure IV, ReSi, and Re_2Si. With these structures, all as-cast samples' powder patterns can be indexed and refined. However, the refined lattice parameters are scattered across the series of samples and not very close to those obtained from single crystal refinement (Figure 4). This is firstly because it is difficult to deconvolute the overlapped and coincided peaks as Structure III, IV, and V are close in lattice parameters. And we cannot rule out the possibility that there are other incommensurate structures that have not been identified yet, either. We are continuing to search for new structures through single crystal diffraction with these as-cast samples.

Lattice Parameters from LeBail Refinement

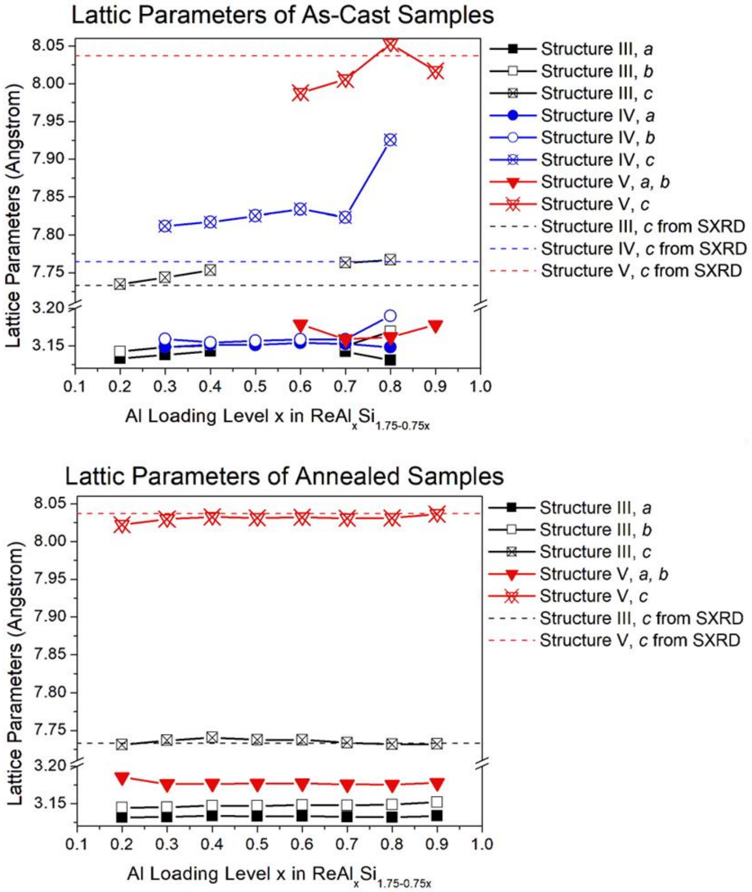

Figure S4. The basic structure lattice parameters of Structure III, IV, and V in all samples obtained from LeBail refinement. The dash lines are the *c* parameters from single crystal XRD (SXRD).

Table S4. The basic structure lattice parameters of Structure III, IV, and V in as-cast samples obtained from LeBail refinement.

Loading Comp. x in ReAl$_x$Si$_{1.75-0.75x}$	Structure III			Structure IV			Structure V	
	a (Å)	*b* (Å)	*c* (Å)	*a* (Å)	*b* (Å)	*c* (Å)	*a* (Å)	*c* (Å)
0.2	3.1332(2)	3.1423(1)	7.7346(4)		–			
0.3	3.1377(2)	3.1484(2)	7.7435(3)	3.1486(3)	3.1592(3)	7.8116(4)	–	
0.4	3.1427(2)	3.1505(3)	7.7532(4)	3.1517(5)	3.1546(5)	7.8171(3)		
0.5		–		3.1512(1)	3.1566(2)	7.8252(3)		
0.6				3.1541(3)	3.1585(3)	7.8339(5)	3.1787(7)	7.988(1)
0.7	3.142(2)	3.150(2)	7.763(2)	3.1528(8)	3.1587(7)	7.823(1)	3.1588(3)	8.006(1)
0.8	3.1306(7)	3.1691(3)	7.7668(5)	3.1482(4)	3.1902(4)	7.9255(6)	3.1618(2)	8.0532(6)
0.9		–			–		3.1779(2)	8.0171(6)

Table S5. The basic structure lattice parameters of Structure III and V in annealed samples obtained from LeBail refinement.

Loading Comp. x in $ReAl_xSi_{1.75-0.75x}$	Structure III			Structure V	
	a (Å)	*b* (Å)	*c* (Å)	*a* (Å)	*c* (Å)
0.2	3.1314(2)	3.1444(2)	7.7310(5)	3.1853(3)	8.022(1)
0.3	3.13215(8)	3.14531(7)	7.7369(2)	3.1761(1)	8.0298(4)
0.4	3.13379(8)	3.14724(8)	7.7405(2)	3.1766(1)	8.0327(3)
0.5	3.1330(1)	3.1470(1)	7.7376(3)	3.17670(8)	8.0314(3)
0.6	3.13303(9)	3.14812(9)	7.7374(2)	3.17708(7)	8.0321(3)
0.7	3.1323(1)	3.1477(1)	7.7338(2)	3.17586(6)	8.0309(2)
0.8	3.1318(1)	3.1487(1)	7.7316(3)	3.17560(4)	8.0311(2)
0.9	3.1334(2)	3.1519(2)	7.7320(3)	3.17765(4)	8.0364(1)

Epitaxial Intergrowth between Structure IV and V

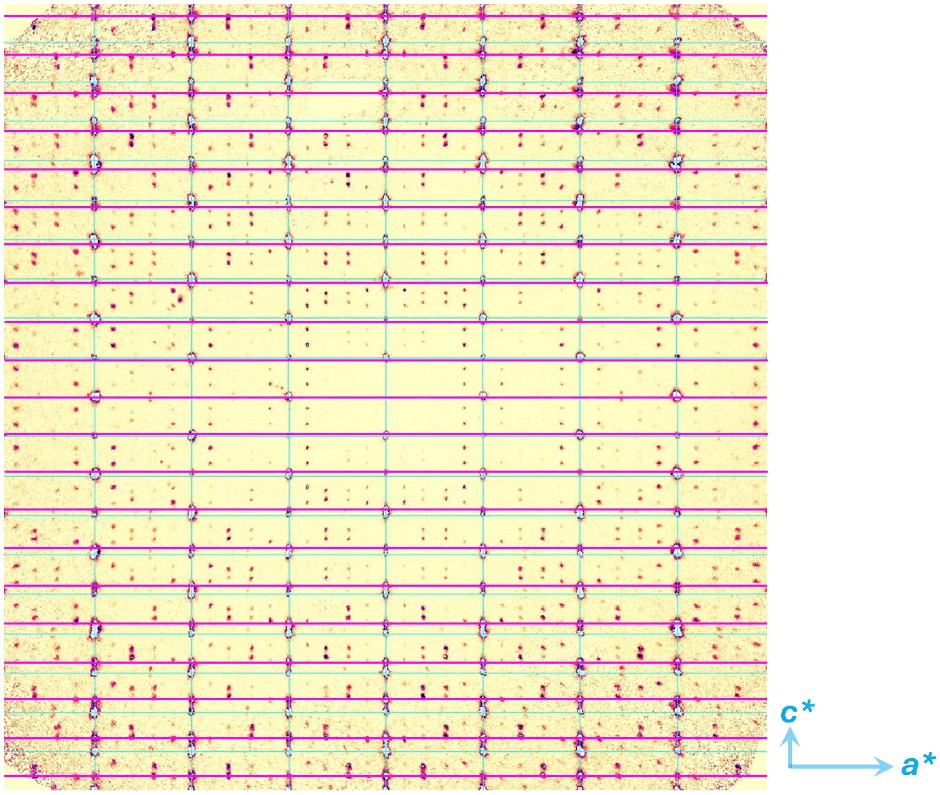

Figure S5. The ($h1l$) precession image of a "single crystal" of Structure IV showing actually the epitaxial intergrowth of Structure IV (cyan) and V (magenta), which share equal ***a****** but different ***c****.

Details of Computational Model Structures

Table S6. The structural details of computational structure Model 1.

Model 1	*Pm* (6), *a* = 15.6408 Å, *b* = 3.1380 Å, *c* = 16.6557 Å, *β* = 112.0031°								
Atom	Wyck.	*x*	*y*	*z*	Atom	Wyck.	*x*	*y*	*z*
Re1	1*a*	0.0054	0	0.0062	Si9	1*b*	0.3295	1/2	0.5750
Re2	1*a*	0.8043	0	0.5047	Si10	1*b*	0.5337	1/2	0.5843
Re3	1*a*	0.6003	0	0.0010	Si11	1*b*	0.3334	1/2	0.0819
Re4	1*a*	0.8018	0	0.0021	Si12	1*b*	0.1305	1/2	0.5745
Re5	1*a*	0.5986	0	0.5042	Si13	1*b*	0.1333	1/2	0.0851
Re6	1*a*	0.4015	0	0.0057	Si14	1*b*	-0.0672	1/2	0.5828
Re7	1*a*	0.4061	0	0.5075	Si15	1*b*	-0.0690	1/2	0.0765
Re8	1*a*	0.1994	0	0.0054	Si16	1*b*	0.7313	1/2	0.0767
Re9	1*a*	0.1989	0	0.4968	Si17	1*a*	0.8672	0	0.6690
Re10	1*a*	-0.0004	0	0.5060	Si18	1*a*	0.6678	0	0.1667
Re11	1*b*	0.7999	1/2	0.7447	Si19	1*a*	0.4701	0	0.6746
Re12	1*b*	0.5959	1/2	0.2456	Si20	1*a*	0.6689	0	0.6749
Re13	1*b*	0.3936	1/2	0.7417	Si21	1*a*	0.4664	0	0.1668
Re14	1*b*	0.6008	1/2	0.7521	Si22	1*a*	0.2655	0	0.6647
Re15	1*b*	0.4015	1/2	0.2473	Si23	1*a*	0.2713	0	0.1763
Re16	1*b*	0.1995	1/2	0.7430	Si24	1*a*	0.0687	0	0.6698
Re17	1*b*	0.1997	1/2	0.2494	Si25	1*a*	0.0684	0	0.1732
Re18	1*b*	-0.0035	1/2	0.7496	Si26	1*a*	0.8680	0	0.1717
Re19	1*b*	0.0021	1/2	0.2465	Si27	1*a*	-0.0806	0	0.8435
Re20	1*b*	0.7958	1/2	0.2410	Si28	1*a*	0.5304	0	0.3341
Si1	1*b*	0.7841	1/2	0.8824	Si29	1*a*	0.3540	0	0.8410
Si2	1*b*	0.5193	1/2	0.8813	Si30	1*a*	0.2776	0	0.3702
Si3	1*a*	0.6505	0	0.8770	Si31	1*b*	0.1468	1/2	0.3725
Si4	1*b*	0.4441	1/2	0.4082	Si32	1*a*	0.0116	0	0.3659
Si5	1*b*	0.0638	1/2	-0.0822	Al1	1*b*	0.6666	1/2	0.4155
Si6	1*b*	0.8794	1/2	0.4074	Al2	1*b*	0.2715	1/2	-0.0853
Si7	1*b*	0.7313	1/2	0.5804	Al3	1*a*	0.7390	0	0.3336
Si8	1*b*	0.5292	1/2	0.0749	Al4	1*a*	0.1346	0	0.8348

Table S7. The structural details of computational structure Model 2.

Model 2	$Pmc2_1$ (26), a = 3.1380 Å, b = 7.7295 Å, c = 31.2483 Å			
Atom	Wyck.	x	y	z
Re1	2a	0	-0.0002	-0.0023
Re2	2a	0	0.0138	0.0987
Re3	2a	0	0.0119	0.1972
Re4	2a	0	0.0088	0.3003
Re5	2a	0	-0.0142	0.3973
Re6	2b	1/2	0.4936	0.0474
Re7	2b	1/2	0.4909	0.1481
Re8	2b	1/2	0.4905	0.2499
Re9	2b	1/2	0.5081	0.3470
Re10	2b	1/2	0.5055	0.4483
Si1	2a	0	0.3423	-0.0021
Si2	2a	0	0.3499	0.0980
Si3	2a	0	0.3400	0.1970
Si4	2a	0	0.3314	0.3011
Si5	2a	0	0.2617	0.4332
Si6	2a	0	0.3358	0.7982
Si7	2a	0	0.3501	0.8983
Si8	2b	1/2	0.1540	0.0475
Si9	2b	1/2	0.1675	0.1473
Si10	2b	1/2	0.1650	0.2482
Si11	2b	1/2	0.1831	0.3576
Si12	2b	1/2	0.1799	0.6395
Si13	2b	1/2	0.1641	0.8486
Si14	2b	1/2	0.1552	-0.0516
Si15	2b	1/2	0.2520	0.4989
Si16	2a	0	0.2671	0.5656
Al1	2a	0	0.3277	0.6952
Al2	2b	1/2	0.1642	0.7478
